# Performing Automatic Identification and Staging of Urothelial Carcinoma in Bladder Cancer Patients Using a Hybrid Deep-Machine Learning Approach

**DOI:** 10.3390/cancers15061673

**Published:** 2023-03-08

**Authors:** Suryadipto Sarkar, Kong Min, Waleed Ikram, Ryan W. Tatton, Irbaz B. Riaz, Alvin C. Silva, Alan H. Bryce, Cassandra Moore, Thai H. Ho, Guru Sonpavde, Haidar M. Abdul-Muhsin, Parminder Singh, Teresa Wu

**Affiliations:** 1 Department Artificial Intelligence in Biomedical Engineering, Friedrich-Alexander-Universität Erlangen-Nürnberg, 91054 Erlangen, Germany; 2 Department of Radiology, Mayo Clinic, Phoenix, AZ 85054, USA; 3 Division of Hematology and Oncology, Mayo Clinic, Phoenix, AZ 85054, USA; 4 Dana Farber Cancer Institute, Harvard Medical School, Boston, MA 02215, USA; 5 Department of Internal Medicine, Mayo Clinic, Phoenix, AZ 85054, USA; 6 ASU-Mayo Center for Innovative Imaging, School of Computing and Augmented Intelligence, Arizona State University, Tempe, AZ 85281, USA

**Keywords:** bladder cancer, urothelial carcinoma, lymph node metastasis, deep learning, computed tomography (CT) imaging, machine learning

## Abstract

**Simple Summary:**

Early and accurate bladder cancer staging is important as it determines the mode of initial treatment. Non-muscle invasive bladder cancer (NMIBC) can be treated with transurethral resection whereas muscle invasive bladder cancer (MIBC) requires neoadjuvant chemotherapy with subsequent cystectomy as indicated. Our hybrid machine/deep learning model demonstrates improved accuracy of bladder cancer staging by CECT using a hybrid machine/deep learning model which will facilitate appropriate clinical management of the patients with bladder cancer, ultimately improving patient outcome.

**Abstract:**

Accurate clinical staging of bladder cancer aids in optimizing the process of clinical decision-making, thereby tailoring the effective treatment and management of patients. While several radiomics approaches have been developed to facilitate the process of clinical diagnosis and staging of bladder cancer using grayscale computed tomography (CT) scans, the performances of these models have been low, with little validation and no clear consensus on specific imaging signatures. We propose a hybrid framework comprising pre-trained deep neural networks for feature extraction, in combination with statistical machine learning techniques for classification, which is capable of performing the following classification tasks: (1) bladder cancer tissue vs. normal tissue, (2) muscle-invasive bladder cancer (MIBC) vs. non-muscle-invasive bladder cancer (NMIBC), and (3) post-treatment changes (PTC) vs. MIBC.

## 1. Introduction

Bladder cancer imaging can be misleading. Findings such as perivesical fat stranding, hydronephrosis, focal bladder wall thickening, or a small bladder lesion may be wrongly perceived as a more advanced stage of bladder cancer. It is common to see small lymph nodes in the pelvis post transurethral resection of bladder tumor (TURBT) [1,2,3] or at the time of diagnosis [4]. These otherwise non-significant lymph nodes may harbor metastasis, which in turn might be difficult to decipher relying solely on visual inspection from CT scans [5,6]. Furthermore, bladder cancer is a heterogeneous disease with an extremely varied range of case-specific diagnoses [7,8]. From low-grade tumors such as $T_{a}$ (noninvasive papillary carcinoma) and $T_{is}$ (tumor in situ or “flat lesion”), which require TURBT or less aggressive endoscopic intervention [8], to high-grade muscle-invasive tumors, which require chemotherapy [9,10], bladder cancer diagnosis is highly dependent on the type and stage of the tumor [4,6,11].

Radiomics can provide tools to significantly improve the accuracy of clinical staging by analyzing multiple qualitative features, including but not restricted to texture analysis, raw digital data and deep model-generated embeddings.

Texture analysis helps capture local patterns in images from the intensity information contained within them. Such features are very effective in identifying tissue types from grayscale medical scans. The texture-based features generally used in medical imaging can be broadly categorized into two subdivisions, namely statistical approaches and transformation-based approaches.

First, we will review some of the most commonly used statistical texture features for bladder cancer detection and staging from medical scans. Ref. [12] utilized a set of functional, second-order statistical and morphological features to perform staging between T1 and T2 types of bladder cancer from MRI scans. The second-order statistical feature extraction approach included a total of 25 GLCM features and 16 gray level run length matrix (GLRLM) features obtained from 42 bladder MRIs (21 T1, 21 T2) post ROI segmentation, yielding an accuracy, sensitivity and specificity of 95.24% and an AUC of 98.64%. Furthermore, the authors also performed an extensive comparison between their proposed model and two state-of-the-art approaches, namely [13,14]. Ref. [15] makes use of LBP and GLCM features to perform primary tumor staging of bladder cancer into two groups: (1) tumor stage and primary tumor located completely within the bladder; (2) tumor stage and primary tumor extending outside the bladder. The SVM classifier, which was trained on T2-weighted MRI scans from 65 bladder cancer patients all with stage 1, reported an AUC of 80.60%. Ref. [16] performs prediction of recurrence and progression of urothelial carcinoma from a dataset of 42 patients—13 without recurrence, 14 with recurrence but not progression, and 15 with progression. Features extracted using LBP and local variance, after classification using the RUSBoost classifier, provided an accuracy of 70% and sensitivity of 84%. Ref. [17] utilizes LBP and GLCM features to classify the invasiveness of bladder cancer. The dataset comprised T2-weighted MRI scans from 65 preoperative bladder cancer patients followed by radical cystectomy. The proposed model reported a patient-level sensitivity of 74.20%, specificity of 82.40%, accuracy of 78.50% and AUC of 80.60%. Ref. [18] performed survival prediction of bladder urothelial carcinoma (BLCA) from CECT scans by utilizing LBP, wavelet and GLCM features. The dataset comprised scans from 62 bladder cancer patients with stages of urothelial carcinoma. The radiomics features extracted from the CECTs were used in combination with RNA-seq data for a complete radiogenomics signature, which in turn helped predict the survival of the patients. This study exhibits the applicability of radiomics and transcriptomics data in predicting BLCA survival. However, owing to the sheer small size of the dataset, the authors think that the model needs to be validated on a larger set of samples for a more foolproof analysis. The literature exhibits that statistical texture analysis is not only fast and easy to implement but also very effective in performing classification, staging and segmentation of bladder cancer from medical images.

Transformation-based approaches such as Fourier and Gabor wavelet transform are very effective in learning textural patterns from images and are therefore a popular option among medical imaging researchers. Ref. [19] utilized Gabor features to perform carcinoma cell classification from biopsy images associated with 14 distinct cancer types, scanned from 14 different patients. An SVM classifier with a radial basis function (RBF) kernel was subsequently trained on these features. The SVM classifier provides the highest cross-validation accuracy of 99.20% with a Gabor window size of 400 pixels and an image magnification of 10. Ref. [20] makes use of 2D Fourier-based features to identify cancer from 182 optical coherence tomography (OCT) scans obtained from 21 patients who were identified as high risk of having transitional cell carcinoma (TCC) and from 68 different areas of the bladder. The task was two-fold: (1) to perform classification between non-cancerous, dysplasia, carcinoma in situ (CIS), and papillary lesions; (2) to predict the invasiveness of the lesion. Other than 2D Fourier transform, the authors also extracted four different statistical feature extraction approaches, resulting in a total of 74 features obtained from the raw OCT images. A simple cross-correlation-based filter with a correlation threshold of 0.85 was employed for feature selection, which resulted in a final set of nine selected features. The decision tree classifier was utilized to finally perform classification on the selected feature set. The authors reported a non-cancerous versus cancerous classification sensitivity of 92.00% and specificity of 62.00%. Ref. [21], which has been summarized earlier, reports that GLCM and GLDM perform better than Fourier transform-based feature extractors in differentiating between tumors and peritumoral fat tissues.

From the literature reviewed above, it is evident that texture analysis is an effective approach when performing classification tasks on medical imaging, in general, and histologic analysis in particular.

Recently, deep learning-based models have emerged and gained popularity among researchers owing to their automatic feature extraction capabilities. Convolutional neural networks (CNNs) are the most commonly used type of deep models and a very popular framework when performing classification tasks on imaging-based applications, in general, and radiological data in particular. The authors of [22] designed a set of nine CNN-based models for the classification of MIBC and NMIBC from contrast-enhanced CT (CECT) images. The dataset comprised 1200 CT scans obtained from 369 patients undergoing radical cystectomy. A total of 249 out of these patients had NMIBC, while the remaining 120 had MIBC. The CNN model was pre-trained on the ImageNet dataset in order to improve classification performance. The model with the highest AUC on the test set was obtained using the VGG16 algorithm—with an AUC of 99.70%, accuracy of 93.90%, sensitivity of 88.90%, specificity of 98.90%, precision of 98.80% and negative predictive value of 89.90%. In contrast, the authors of [13] made use of Haralick features, which are a variant of GLCM, to identify muscular invasiveness in MRI scans containing a total of 118 volumes of interest (VOI) obtained from 68 patients—34 volumes labeled non-muscle-invasive bladder cancer (NMIBC), and 84 labeled muscle-invasive bladder cancer (MIBC). The final SVM classifier obtained an AUC of 86.10% and Youden index of 71.92%.

Ref. [21] performs classification between bladder cancers with and without response to chemotherapy from a set of CT scans obtained before and after treatment. The authors have reviewed three different models and their capabilities in performing classification: (1) a deep learning-convolutional neural network-based model (DL-CNN); (2) a more deterministic radiomics feature-based classifier (RF-SL); (3) an intermediate model that extracts radiomics features from image patterns (RF-ROI). The training dataset comprised 82 patients having 87 bladder cancers, scanned pre- and post-chemotherapy. The test set comprised 41 patients with 43 cancers. The radiomics feature-based model (RF-SL) performed the best with an AUC of 77.00%, while the two radiologists reported AUCs of 76.00% and 77.00%, respectively.

Ref. [23] proposed a CNN-based model for performing classification between low- and high-stage bladder cancer. The training dataset comprised 84 bladder cancer CR urography (CTU) images obtained from 76 patients (43 CTUs contained low-stage cancer, while 41 contained high-stage cancer). The test set consisted of 90 bladder CTUs obtained from 86 patients. The CNN classifier had a test set prediction accuracy of 91.00%, which the authors claim is higher as compared to texture-based classification using SVM on the same dataset (which had a prediction accuracy of 88.00%). In comparison, ref. [21] extracted GLCM- and histogram-based features from apparent diffusion coefficient (ADC) and diffusion-weighted images (DWI) to perform bladder cancer grading. A total of 61 patients were scanned for this study, 32 out of whom were in low-grade and the remaining 29 in high-grade classes. A combination of 102 GLCM and histogram features were initially extracted, out of which 47 were finally selected using the Mann–Whitney U-test, and an SVM classifier was used to perform classification between high- and low-grade bladder cancer with an accuracy of 82.90%. Ref. [21] extracted histogram and GLCM features to perform classification between high-grade and low-grade bladder cancer scans from a set of diffusion-based MRIs obtained from 61 bladder cancer patients (32 of them having low-grade and 29 having high-grade bladder cancer), yielding an accuracy of 82.90% and area under the curve (AUC) of 86.10%. Ref. [24] made use of GLCM, wavelet filter and Laplacian of Gaussian filter to extract features from a small dataset of 145 patients to perform grading on bladder cancer CT scans. Out of these 145 scans, 108 were used to train the model, and the remaining 37 were used to perform validation. The model provided an accuracy of 83.80% on the validation set.

From the literature reviewed above, it is evident that neural network-based classifiers are more effective than texture analysis when performing identification and staging of bladder cancer from medical imaging alone.

The authors of [25] claim that feature extraction when governed by domain knowledge performs better than CNN-based classifiers that are capable of automatic feature generation. The task in this case was to classify two early stages of bladder cancer that are histologically difficult to differentiate, namely Ta (non-invasive) and T1 (superficially invasive). The dataset comprised a total of 1177 bladder scans—460 non-invasive, 717 superficially invasive. CNN classifiers achieved the highest accuracy of 84.00%, performing considerably poorer than supervised machine learning classifiers that were trained on manually extracted features. The aforementioned literature on CNNs comprises end-to-end models, where both training and testing are performed on the same dataset. The problem with such an approach is that deep neural networks require large quantities of training data in order to avoid the pervasive issue of over-fitting. As a substitute to end-to-end deep models, researchers make use of a concept called “transfer learning”, where the neural network is first pre-trained on a large dataset such as ImageNet, and the learned weights are subsequently fine-tuned on the small target data. Since we were using a small dataset of 200 CT scans for this study, we decided to make use of transfer learning to improve classification results and alleviate overfitting. A 71-layer ResNet-18 model pre-trained on the publicly available ImageNet dataset was utilized to extract features from the bladder scans. The extracted features, after feature selection using a combination of supervised and unsupervised techniques, were finally used to perform classification by five different machine learning classifiers, namely k-nearest neighbor (KNN), support vector machine (SVM), linear discriminant analysis (LDA), decision tree (DT) and naive Bayes (NB).

## 2. Materials and Methods

**Figure 1:**
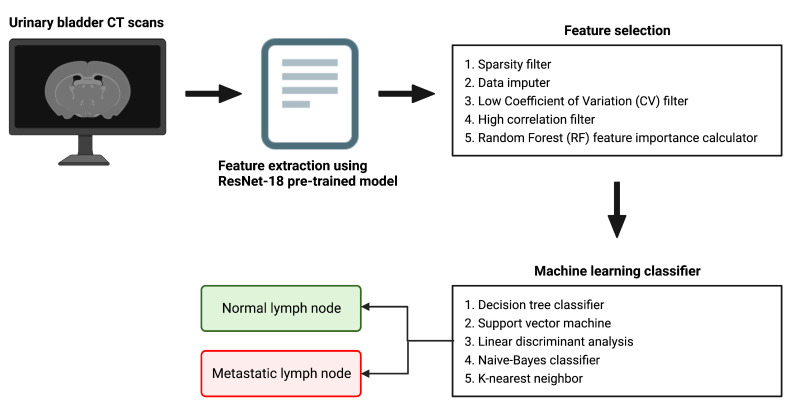
A schematic representation of the overall workflow.

Figure 1 provides a pictorial depiction of the entire workflow, starting from the raw bladder scans to the prediction labels obtained after classification. The proposed methodology comprises feature extraction, feature selection, and finally classification. In this hybrid approach, we extract feature vectors from the images using the trained model weights from the last pooling layer of the five most widely used pre-trained deep models, namely AlexNet, GoogleNet, InceptionV3, ResNet-50 and XceptionNet. We subsequently employ our feature selection algorithm on this feature vector. Finally, we use five machine learning classification algorithms on the selected feature set, namely k-nearest neighbor (KNN), naive Bayes (NB), support vector machine (SVM), linear discriminant analysis (LDA) and decision tree (DT).

### 2.1. Feature Extraction Using Pre-Trained Deep Models

Five popular neural network-based deep models, namely AlexNet, GoogleNet, InceptionV3, ResNet-50 and XceptionNet, all pre-trained on the ImageNet dataset [26], were trained using our bladder CT scan data to fine-tune the model parameters. The trained weights from the last pooling layer of each of these models were extracted and subsequently used as feature descriptors. Table 1 provides a comprehensive description of each of these models—including information regarding the pooling layer and the size of the extracted feature vector.

### 2.2. Feature Selection Mechanism

**Figure 2:**
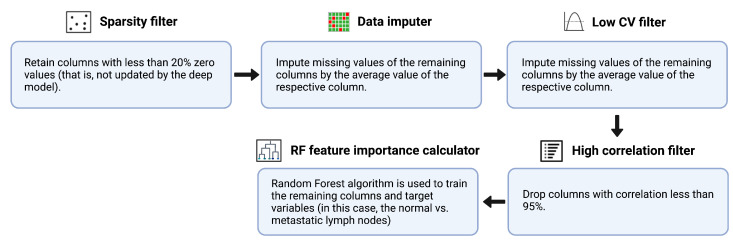
A pictorial representation of the feature selection procedure.

An ensemble feature selection technique was used to select the most important features from the originally extracted feature vector—which was obtained using five different pre-trained models, namely AlexNet, GoogleNet, Inception V3, ResNet-50 and XceptionNet, for performing the classification of normal vs. metastatic lymph nodes. First, we make use of a “sparsity filter” to remove the features that were not updated by the deep model. Through this step, we not only exclude features that have low variance but also automatically remove those that are less likely to have an impact on the classification process. Next, we utilize the “data imputer” to impute the unchanged values of the remaining columns by the mean value of the respective column. Subsequently, we made use of the “low coefficient of variation (CV) filter” to drop features with a CV value of less than 0.1. CV, or standard deviation normalized by mean, is a measure of information content; a lower CV indicates features with a lower normalized variance. Furthermore, in the next step, a correlation matrix was generated, which contains information regarding the cross-correlation values between every pair of features. For each pair with >95% correlation, the one with a lower correlation to the output is dropped. Finally, we employed random forest, a boosted decision tree algorithm, to train the remaining columns and target variables for feature importance score generation. The number of trees was set to a default value of 100, and the Gini index was used as the metric for calculating feature importance. The first four steps were unsupervised (statistical approaches used to perform selection on features alone, not labels); the last feature importance calculation step was supervised (relationship between dependent and independent variables critical in determining feature selection). Figure 2 provides a schematic depiction of the feature selection algorithm.

### 2.3. Machine Learning-Based Classification

The important features obtained using the feature selection algorithm were used as inputs into the machine learning classifier. The three classification tasks that were performed were: (1) normal vs. bladder cancer, (2) NMIBC vs. MIBC, (3) post-treatment changes (PTC) vs. MIBC. A 10-fold cross-validation was utilized to evaluate the prediction performance of the proposed model. The samples were randomly re-arranged, then the dataset was split into 10 equal divisions. Nine of these divisions, at every iteration, were used for training; and the remaining was one used for testing. This process was repeated for 10 iterations, each time the test set being a different group. The prediction evaluation metrics calculated across the ten iterations were averaged and reported. Classification was performed using five different machine learning classifiers, namely k-nearest neighbor (KNN), naive Bayes (NB), support vector machine (SVM), linear discriminant analysis (LDA) and decision tree (DT).

### 2.4. Evaluation Metrics

In order to evaluate the efficacy of the classification model, the following five metrics were used:

(1) $$Accuracy=\frac{TP+TN}{TP+TN+FP+FN}$$

(2) $$Sensitivity=\frac{TP}{TP+FN}$$

(3) $$Specificity=\frac{TN}{TN+FP}$$

(4) $$Precision=\frac{TP}{TP+FP}$$

(5) $$F1=\frac{2\times Precision\times Sensitivity}{Precision+Sensitivity}$$

where *TP* is the # of true positives, *TN* is the # of true negatives, *FP* is the # of false positives and *FN* is the # of false negatives.

Since our dataset is small and highly imbalanced, accuracy, precision and recall are not ideal in representing the classification performance of the models. Therefore, the F1-score was used to determine the overall effectiveness of the classifiers. While accuracy represents the overall percentage of correctly classified samples, precision represents the percentage of identified samples where the condition actually exists, and recall represents the proportion of samples with the condition that have been correctly diagnosed, none of them corrects for data imbalance. The F1-score, which is the harmonic mean of precision and recall, has been employed to rank classifiers in terms of diagnostic performance for this particular study.

## 3. Software and Tools

MATLAB version R2021b (developed by MathWorks, Massachussets USA) was used for the purpose of feature extraction and machine learning-based classification (The Deep Learning Toolbox was used to train the five pre-trained deep models, namely AlexNet, GoogleNet, InceptionV3, ResNet50 and XceptionNet. The Statistics and Machine Learning Toolbox was used to train the four machine learning-based classifiers, namely naive Bayes, support vector machine, linear discriminant analysis and decision tree).

**Figure 3:**
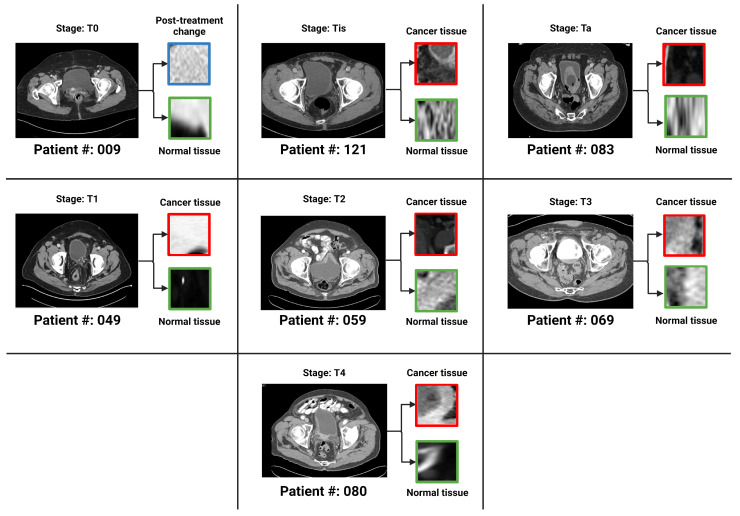
One bladder CT scan per stage (along with the corresponding regions of interest that were used in the various classification tasks) have been provided. The 7 stages of urothelial carcinoma analyzed in the study are: $T_{a}$, $T_{is}$, $T_{0}$, $T_{1}$, $T_{2}$, $T_{3}$ and $T_{4}$ ($T_{0}$ has not been shown in the figure because $T_{0}$ represents a stage where the tissue of interest shows no evidence of malignancy).

**Figure 4:**
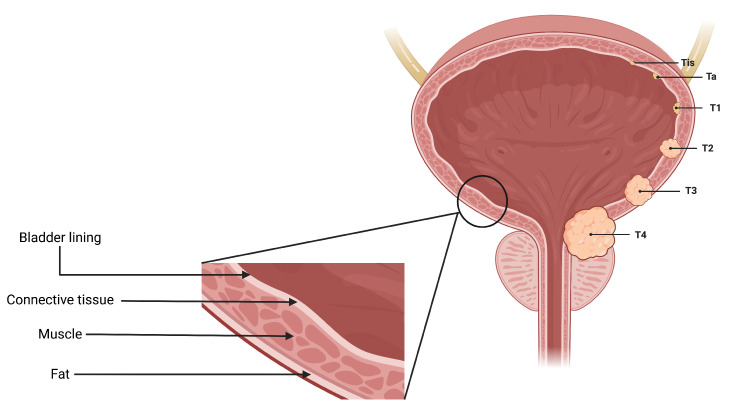
A pictorial representation of the 7 stages of urothelial carcinoma analyzed in the study ($T_{0}$ has not been shown in the figure because $T_{0}$ represents a stage where the tissue of interest shows no evidence of malignancy).

ImageJ (developed by National Institutes of Health, Maryland USA) and RadiAnt Dicom Viewer (open source application) were used to analyze the CT images. BioRender was used to generate all the illustrations in the paper (Figures 1–4).

**Figure 5:**
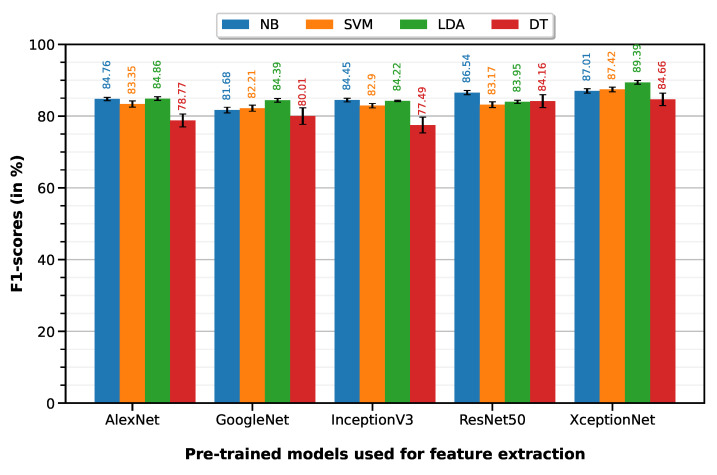
F1-scores of the 10-fold machine learning classifiers on features extracted from each of the five pre-trained deep models.

**Figure 6:**
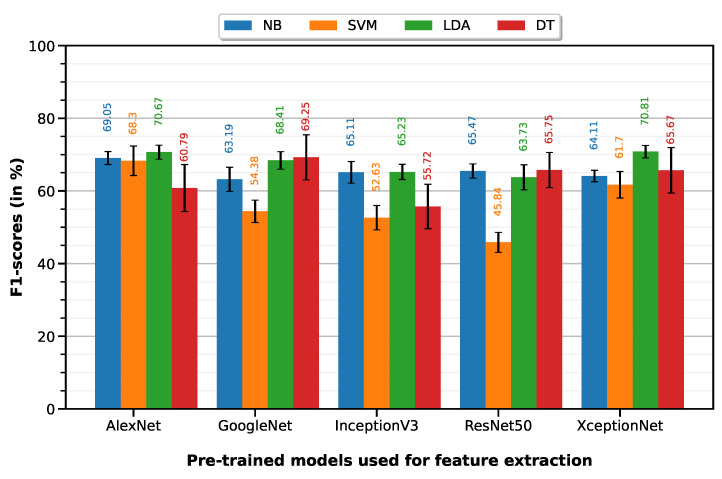
F1-scores of the 10-fold machine learning classifiers on features extracted from each of the five pre-trained deep models.

**Figure 7:**
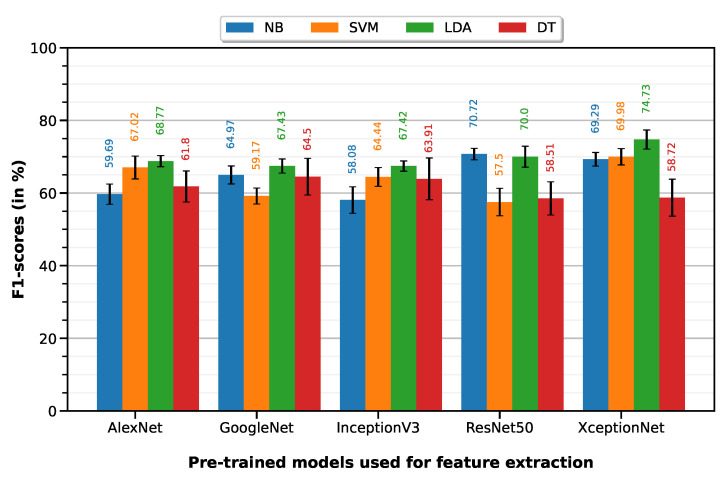
F1-scores of the 10-fold machine learning classifiers on features extracted from each of the five pre-trained deep models.

Python 3 (open source programming language) was used to program the feature selection workflow and generate the plots containing the F1-scores per model per classifier (Figures 5–7).

## 4. Dataset

A urothelial carcinoma dataset was provided by Mayo Clinic, Arizona. The dataset contained de-identified grayscale CT scans obtained from patients who were imaged before radical cystectomy and pelvic lymph node dissection as part of a trial. The location of each bladder mass was confirmed, and labels of the preoperative CT data were generated. The labels were “cancer” (meaning malignant cells) and “normal” (normal bladder wall).

There were a total of 100 CT scans of the pelvis with intravenous contrast images visualizing the bladder, obtained from 100 patients (one image captured per patient). Each scan had 2 masks (normal bladder wall and bladder cancer) manually annotated by encompassing both the entire region of biopsy-proven malignancy and the normal-appearing bladder by two radiologists familiar with bladder imaging, which were used to extract the respective regions of interest (ROI)—therefore resulting in 200 ROIs (100 normal tissue and 100 abnormal tissue). These ROIs were used as input for classification instead of the entire image. The patients were distributed across seven bladder cancer stages, namely $T_{a}$, $T_{is}$, $T_{0}$, $T_{1}$, $T_{2}$, $T_{3}$ and $T_{4}$. Figure 3 provides an axial CT scan with IV contrast, along with its corresponding region of interest (ROI) on the bladder wall, pertaining to each of the seven stages. $T_{0}$ represents a stage where the tissue of interest shows no evidence of malignancy, possibly but not necessarily following tumor resection and/or chemotherapy. This indicates carcinoma in situ, where the malignant cells only involves the innermost lining of the bladder wall. $T_{1}$ represents a stage where malignant cells involve the connective tissue beyond the innermost lining without involvement of the bladder muscle. $T_{2}$ represents the malignant spread of tumor involving the bladder muscle. $T_{3}$ represents a stage where malignant mass spreads outside the confines of bladder muscle with involvement of the perivesical fat. $T_{4}$ stage indicates the spread of tumor beyond the bladder with involvement of abdominal/pelvic wall and/or nearby organs. $T_{a}$, $T_{is}$ and $T_{1}$ stages are classified as non-muscle-invasive bladder cancer (NMIBC); $T_{2}$, $T_{3}$ and $T_{4}$ stages are classified as muscle-invasive bladder cancer (MIBC) [4,27]. Figure 4 is a pictorial depiction of the different stages of bladder cancer. As is evident from Figure 3, the stages are difficult to distinguish on visual inspection—thereby making it suitable for classification using AI-based models. Table 2 provides a summary of the number of patients per stage.

## 5. Results

The proposed model was used to perform three different classification tasks during the course of this study: (1) normal vs. bladder cancer; (2) NMIBC vs. MIBC; (3) post-treatment changes (PTC) vs. MIBC. In this section, for each of the three tasks, the best classification performances corresponding to each of the five pre-trained deep model-based features have been presented. The class-wise number of ROIs used for each of the individual tasks has also been summarized.

### 5.1. Normal vs. Cancer

Normal vs. cancer classification was performed with 10-fold cross-validation on a dataset of 165 ROIs (100 normal, 65 cancer)—35 T0 images were not relevant because they represent post-treatment changes (PTC) and not cancer. The LDA classifier on XceptionNet-based features provides the best performance with an accuracy of 86.07%, sensitivity of 96.75%, specificity of 69.65%, precision of 83.07% and F1-score of 89.39%. Table 3 summarizes the classification performances of the 10-fold machine learning classifiers on features extracted from each of the five pre-trained deep models (results visualized on the associated Figure 5 bar plot).

### 5.2. NMIBC vs. MIBC

NMIBC vs. MIBC classification was performed with 10-fold cross-validation on a dataset of 65 ROIs (24 NMIBC, 41 MIBC). The LDA classifier on XceptionNet-based features provides the best performance with an accuracy of 79.72%, sensitivity of 66.62%, specificity of 87.39%, precision of 75.58% and F1-score of 70.81%. Table 4 summarizes the classification performances of the 10-fold machine learning classifiers on features extracted from each of the five pre-trained deep models (results visualized on the associated Figure 6 bar plot).

### 5.3. Post-Treatment Changes (PTC) vs. MIBC

PTC vs. MIBC classification was performed with a 10-fold cross-validation on a dataset of 76 ROIs (35 PTC, 41 MIBC). LDA classifier on XceptionNet-based features provided the best performance with an accuracy of 74.96%, sensitivity of 80.51%, specificity of 70.22%, precision of 69.78% and F1-score of 74.73%. Table 5 summarizes the classification performances of the 10-fold machine learning classifiers, on features extracted from each of the five pre-trained deep models (results visualized on associated Figure 7 bar plot).

## 6. Discussion

Optimal management of bladder cancer requires a multidisciplinary approach, with tumor staging an important prognostic factor that determines the mode of initial treatment. Cystoscopy examination, together with a biopsy, remains the primary mode of tumor detection and clinical staging in patients with suspected bladder cancer. CT is often the first exam modality for bladder cancer detection due to its wide availability and minimal associated complication. However, the CT findings are nonspecific with limited accuracy. At least one article [28] reported the accuracy of CT evaluation for bladder cancer as low as 49%.

Differentiating non-muscle-invasive bladder cancer (NMIBC), consisting of Ta, Tis, and T1 stages, from muscle-invasive bladder cancer (MBIC), consisting of $T_{2}$-$T_{4}$4 stages, is important as MIBC is more likely to spread to lymph nodes and other organs requiring radical cystectomy with or without systemic chemotherapy [29]. In contrast, NMBIC has low risk of recurrent disease but can be effectively treated with intravesical chemotherapy, immunotherapy, and transurethral resection of bladder tumor (TURBT) [30]. Early differentiation of two bladder cancer stages is critical for the appropriate utilization of medical resources and optimization of targeted treatment. Therefore, accurate initial staging on the CT exam is crucial for therapeutic decision-making [31].

While histopathologic cancer detection using pre-trained neural network-based models is mainstream, in this project, we were faced with the additional issues of imbalanced data and limited sample size. In comparison to [32], where the number of image samples was 1350, and the number of classification categories was 2 (1200 bladder cancer tissues, 1150 normal tissues), we had a total of only 100 tissue scans distributed across 7 stages of urothelial carcinoma (6 $T_{a}$, 9 $T_{i}s$, 35 $T_{0}$, 9 $T_{1}$, 13 $T_{2}$, 24 $T_{3}$, 4 $T_{4}$). The issue of class-imbalanced samples could not be solved using SMOTE because the sheer lack of samples in some of the categories (especially $T_{a}$ and $T_{4}$) meant that the synthetically generated samples were very similar to the few original ones, and contributed towards decreased variance in the training dataset—therefore resulting in overfitting. Next, the issue of overfitting, which we faced while performing end-to-end classification using pre-trained networks, was tackled by: (1) making use of a combined deep learning-machine learning approach where the trained weights from the pre-trained neural network were then classified using statistical machine learning approaches. (2) An ensemble statistical- and supervised learning-based feature selection approach that helped remove features that were unimportant. Ref. [32], which focused on bladder cancer detection from cytoscopic images alone, reported an accuracy of 86.36% with deep learning-based classifiers and 84.09% with human surgical experts; however, no significant difference was found between the two (*p*-value greater than 0.05).

The LDA classifier on XceptionNet performed best in terms of the F1-score for all three experiments in our study—namely, normal vs. cancer, NMIBC vs. MIBC and PTC vs. MIBC. For normal vs. cancer classification, LDA on XceptionNet had an F1-score of (89.39 ± 0.26)%, which will facilitate clinicians in better detection of lesions because, for histopathologic images in general, and bladder scans in particular, flat and subtle lesions are often missed on visual inspection. For NMIBC vs. MIBC classification, LDA on XceptionNet had an F1-score of (70.81 ± 0.86). For PTC vs. MIBC classification, LDA on XceptionNet had an F1-score of (74.73 ± 1.31)%, which is especially encouraging because there is an unmet demand to develop new non-invasive techniques to assess accurate prediction of recurrence and response to chemotherapy. Currently, patients with bladder cancer require repeat cystoscopies and biopsies of the bladder to assess the response and recurrence of the disease. This procedure is very costly and invasive, with several associated complications, including bladder perforation.

Our study has certain limitations. It is a retrospective design based on a single-center small dataset that may overestimate the diagnostic performance of our model. Therefore, our next step is to extend the diagnostic model on prospective, multi-center datasets with external validation.

## 7. Conclusions

Our model showed a high F1-score, which means that our model indicated a high value for both recall and precision. We used the F1-score to compare our classifiers. We opted for the ResNet-50, whose F1-score was higher among others and ResNet-50 showed the best classification based on the F1-score for all three experiments.

Radiomics-assisted interpretation of CT by radiologists may help more accurately diagnose bladder cancer. This can allow the timely utilization of medical resources and consultation with oncologists and urologists, ultimately improving patients’ clinical outcomes.

## Figures and Tables

**Table 1:** A description of the five pre-trained models used for feature extraction from the bladder scans, namely AlexNet, GoogleNet, InceptionV3, ResNet-50 and XceptionNet. The table contains the total number of layers per model; the last pooling layer from which features were being extracted; the layer number of the last pooling layer; and the length of the extracted feature vector.

Pre-Trained Model	Total No. of Layers in the Network	Last Pooling Layer	Layer No. of the Last Pooling Layer	Feature Vector Length
AlexNet	25	Max Pooling	16	9216
GoogleNet	144	Global Average Pooling	140	1024
InceptionV3	315	Global Average Pooling	312	2048
ResNet-50	177	Global Average Pooling	174	2048
XceptionNet	170	Global Average Pooling	167	2048

**Table 2:** A summary of the number of patients per stage.

$T_{a}$	$T_{\mathrm{is}}$	$T_{0}$	$T_{1}$	$T_{2}$	$T_{3}$	$T_{4}$
6	9	35	9	13	24	4

**Table 3:** Classification performances of the 10-fold machine learning classifiers on features extracted from each of the five pre-trained deep models.

Feature Extractor	Classifier	Accuracy	Sensitivity	Specificity	Precision	F1-Score
AlexNet	NB	0.8053±0.0029	0.8936±0.004	0.6694±0.0054	0.8062±0.0025	0.8476±0.0024
	SVM	0.7835±0.0058	0.8944±0.0056	0.6128±0.0122	0.7805±0.0055	0.8335±0.0043
	LDA	0.7987±0.0037	0.9307±0.0027	0.5957±0.0078	0.7799±0.0034	0.8486±0.0026
	DT	0.7373±0.0113	0.8043±0.0120	0.6342±0.0231	0.7724±0.0114	0.7877±0.0089
GoogleNet	NB	0.7642±0.0049	0.8674±0.0058	0.6054±0.0085	0.7718±0.0040	0.8168±0.0039
	SVM	0.7661±0.0053	0.8921±0.0068	0.5722±0.0084	0.7624±0.0038	0.8221±0.0043
	LDA	0.7899±0.0036	0.9368±0.0038	0.5640±0.0061	0.7678±0.0027	0.8439±0.0027
	DT	0.7562±0.0136	0.8056±0.0167	0.6803±0.0231	0.7954±0.0123	0.8001±0.0116
InceptionV3	NB	0.7988±0.0031	0.9016±0.0042	0.6406±0.0052	0.7942±0.0024	0.8445±0.0025
	SVM	0.7740±0.0042	0.9036±0.0041	0.5746±0.0080	0.7657±0.0036	0.8290±0.0031
	LDA	0.7935±0.0012	0.9096±0.0010	0.6148±0.0019	0.7841±0.0009	0.8422±0.0009
	DT	0.7295±0.0127	0.7690±0.0155	0.6686±0.0211	0.7816±0.0114	0.7749±0.0110
ResNet50	NB	0.8248±0.0039	0.9290±0.0045	0.6646±0.0073	0.8100±0.0034	0.8654±0.0030
	SVM	0.7771±0.0051	0.9092±0.0065	0.5738±0.0081	0.7665±0.0036	0.8317±0.0040
	LDA	0.7862±0.0028	0.9224±0.0043	0.5766±0.0056	0.7702±0.0022	0.8395±0.0022
	DT	0.8079±0.0111	0.8418±0.0135	0.7558±0.0256	0.8424±0.0135	0.8416±0.0089
XceptionNet	NB	0.8395±0.0035	0.8866±0.0048	0.7671±0.0058	0.8542±0.0031	0.8701±0.0030
	SVM	0.8322±0.0047	0.9614±0.0034	0.6335±0.0100	0.8015±0.0045	0.8742±0.0033
	LDA	0.8607±0.0038	0.9675±0.0027	0.6965±0.0094	0.8307±0.0042	0.8939±0.0026
	DT	0.8145±0.0099	0.8453±0.0134	0.7672±0.0173	0.8486±0.0095	0.8466±0.0086

**Table 4:** Classification performances of the 10-fold machine learning classifiers on features extracted from each of the five pre-trained deep models.

Feature Extractor	Classifier	Accuracy	Sensitivity	Specificity	Precision	F1-Score
AlexNet	NB	0.7768±0.0067	0.6746±0.0117	0.8366±0.0089	0.7079±0.0112	0.6905±0.0090
	SVM	0.7709±0.0143	0.6692±0.0244	0.8305±0.0147	0.6989±0.0214	0.6830±0.0203
	LDA	0.7963±0.0065	0.6650±0.0125	0.8732±0.0076	0.7547±0.0114	0.7067±0.0097
	DT	0.7160±0.0215	0.6008±0.0445	0.7834±0.0286	0.6223±0.0329	0.6079±0.0324
GoogleNet	NB	0.7480±0.0108	0.5867±0.0202	0.8424±0.0124	0.6864±0.0178	0.6319±0.0166
	SVM	0.7238±0.0088	0.4462±0.0158	0.8863±0.0107	0.6984±0.0215	0.5438±0.0154
	LDA	0.7758±0.0069	0.6583±0.0157	0.8446±0.0064	0.7127±0.0099	0.6841±0.0120
	DT	0.7740±0.0220	0.6925±0.0408	0.8217±0.0239	0.6968±0.0312	0.6925±0.0310
InceptionV3	NB	0.7575±0.009	0.6137±0.0194	0.8417±0.0093	0.6945±0.0139	0.6511±0.0149
	SVM	0.7242±0.0069	0.4163±0.0179	0.9044±0.0033	0.7174±0.0114	0.5263±0.0167
	LDA	0.7698±0.0060	0.5854±0.0134	0.8778±0.0056	0.7373±0.0098	0.6523±0.0105
	DT	0.6909±0.0210	0.5300±0.0409	0.7851±0.0306	0.5963±0.0353	0.5572±0.0306
ResNet50	NB	0.6878±0.0103	0.8013±0.0135	0.6215±0.0149	0.5539±0.0105	0.6547±0.0098
	SVM	0.7240±0.0065	0.3167±0.0115	0.9624±0.0074	0.8343±0.0279	0.4584±0.0138
	LDA	0.7748±0.0098	0.5375±0.0221	0.9137±0.0130	0.7874±0.0220	0.6373±0.0173
	DT	0.7665±0.0159	0.6088±0.0300	0.8588±0.0189	0.7190±0.0287	0.6575±0.0242
XceptionNet	NB	0.7240±0.0094	0.6667±0.0001	0.7576±0.0149	0.6182±0.0146	0.6411±0.0079
	SVM	0.7597±0.0094	0.5258±0.0219	0.8966±0.0085	0.7489±0.0167	0.6170±0.0182
	LDA	0.7972±0.0058	0.6662±0.0100	0.8739±0.0058	0.7558±0.0094	0.7081±0.0086
	DT	0.7403±0.0243	0.6733±0.0379	0.7795±0.0286	0.6446±0.0344	0.6567±0.0313

**Table 5:** Classification performances of the 10-fold machine learning classifiers on features extracted from each of the five pre-trained deep models.

Feature Extractor	Classifier	Accuracy	Sensitivity	Specificity	Precision	F1-Score
AlexNet	NB	0.6695±0.0097	0.5323±0.0172	0.7866±0.0114	0.6806±0.0133	0.5969±0.0139
	SVM	0.6880±0.0139	0.6894±0.0216	0.6868±0.0168	0.6530±0.0145	0.6702±0.0157
	LDA	0.7100±0.0070	0.6934±0.0116	0.7241±0.0121	0.6826±0.0091	0.6877±0.0076
	DT	0.6503±0.0187	0.6160±0.0290	0.6795±0.0270	0.6226±0.0215	0.6180±0.0214
GoogleNet	NB	0.7176±0.0101	0.5689±0.0139	0.8446±0.0149	0.7589±0.0180	0.6497±0.0124
	SVM	0.6592±0.0085	0.5366±0.0128	0.7639±0.0111	0.6602±0.0122	0.5917±0.0110
	LDA	0.7276±0.0072	0.6129±0.0126	0.8256±0.0082	0.7502±0.0094	0.6743±0.0097
	DT	0.6725±0.0237	0.6471±0.0321	0.6941±0.0345	0.6464±0.0288	0.6450±0.0252
InceptionV3	NB	0.6495±0.0150	0.5280±0.0207	0.7532±0.0205	0.6475±0.0214	0.5808±0.0182
	SVM	0.6907±0.0100	0.6094±0.0169	0.7600±0.0121	0.6846±0.0122	0.6444±0.0129
	LDA	0.7083±0.0068	0.6554±0.0086	0.7534±0.0109	0.6945±0.0096	0.6742±0.0070
	DT	0.6589±0.0260	0.6583±0.0383	0.6595±0.0327	0.6238±0.0270	0.6391±0.0287
ResNet50	NB	0.6688±0.0085	0.8689±0.0138	0.4980±0.0125	0.5965±0.0066	0.7072±0.0079
	SVM	0.6197±0.0157	0.5600±0.0251	0.6707±0.0232	0.5930±0.0186	0.5750±0.0189
	LDA	0.6895±0.0094	0.7903±0.0306	0.6034±0.0169	0.6298±0.0069	0.7000±0.0144
	DT	0.6161±0.0207	0.5891±0.0298	0.6390±0.0313	0.5838±0.0239	0.5851±0.0229
XceptionNet	NB	0.6849±0.0087	0.7726±0.0142	0.6100±0.0094	0.6284±0.0075	0.6929±0.0094
	SVM	0.6868±0.0105	0.7931±0.0170	0.5961±0.0102	0.6263±0.0085	0.6998±0.0112
	LDA	0.7496±0.0119	0.8051±0.0193	0.7022±0.0133	0.6978±0.0113	0.7473±0.0131
	DT	0.6253±0.0219	0.5806±0.0320	0.6634±0.0298	0.5967±0.0251	0.5872±0.0254

## Data Availability

Data will be made available under reasonable request.
